# Association of Gut Microbial Genera with Heart Rate Variability in the General Japanese Population: The Iwaki Cross-Sectional Research Study

**DOI:** 10.3390/metabo12080730

**Published:** 2022-08-07

**Authors:** Masaya Tsubokawa, Miyuki Nishimura, Tatsuya Mikami, Mizuri Ishida, Takayoshi Hisada, Yoshinori Tamada

**Affiliations:** 1 Innovation Center for Health Promotion, Graduate School of Medicine, Hirosaki University, Hirosaki City 036-8562, Japan; 2 Health Science Research Center, FANCL Research Institute, Yokohama City 244-0806, Japan; 3 TechnoSuruga Laboratory Co., Ltd., Shizuoka City 424-0065, Japan

**Keywords:** general Japanese population, heart rate variability, gut microbiota, gut microbial diversity, *Lachnospiraceae incertae sedis*

## Abstract

The gut microbiota has become a significant factor associated with health and disease. Although many studies have reported the implications of changes in the gut microbiota on cardiovascular diseases, there are no reports on the relationship between heart rate variability (HRV) and the gut microbiota. Therefore, we investigated the association between gut microbiota abundance and HRV parameters in this cross-sectional study of the general Japanese population. This study included 950 participants of the Iwaki Health Promotion Project who underwent a medical examination in 2019 that included HRV and gut microbiota measurements. At the genus level, multivariate regression analysis showed that higher gut microbial diversity was associated with a higher standard deviation of RR intervals (SDNN). Moreover, a higher SDNN was associated with a higher relative count of *Lachnospiraceae incertae sedis*. *L. incertae sedis* abundance was associated with higher HRV parameters such as SDNN, coefficient of variation of RR intervals, low-frequency component power (LF)/high-frequency component power, and LF. In the general Japanese population, higher gut microbial diversity and *L. incertae sedis* abundance were associated with higher HRV parameters.

## 1. Introduction

The gut microbiota has become a major factor associated with health and disease, and its influence on physiological, behavioral, and cognitive functions of the brain is well recognized. However, the exact mechanisms have not been completely understood [1]. The brain–gut interaction involves the gut microbiota and its metabolic, enteric nervous system, neuroendocrine system, neural-immune system, central nervous system, and autonomic nervous system [2]. Moreover, the abundance of gut microbiota has been suggested to influence homeostasis by changing the properties of autonomic nerve signaling and related neuronal networks [2,3].

The autonomic nervous system is part of the peripheral nervous system and regulates involuntary physiological processes such as sexual arousal, digestion, respiration, blood pressure, and heartbeat [4]. It is also integral in maintaining homeostasis and behavioral functions [5]. The autonomic nervous function is clinically evaluated via heart rate variability (HRV) analysis, an index of autonomic nervous activity related to cardiovascular function. HRV analysis is a non-invasive measurement technique used in various fields [6,7]. In the general population, lower HRV has been associated with cardiovascular disease (CVD) risk and is clinically important [8]. Prevention of CVD is very important as these diseases are among the leading causes of morbidity and mortality in developed countries.

Although many studies have reported that changes in gut microbiota composition are implicated in CVD [9,10,11,12], there are no reports on the relationship between HRV and gut microbiota abundance. Therefore, this study aimed to investigate the association between gut microbiota abundance and HRV parameters in the general Japanese population using data from a medical examination in the Iwaki Health Promotion Project.

## 2. Results

**Figure 1:**
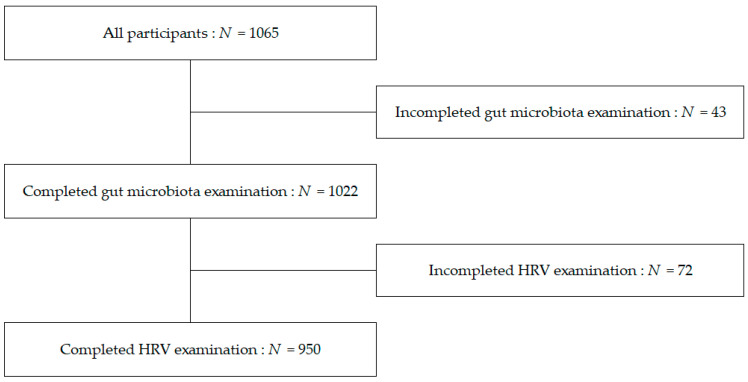
Number of examinations completed.

Figure 1 illustrates the number of examinations completed by study participants. Among the 1065 participants in the Iwaki Health Promotion Project medical examination, 43 did not have their gut microbiota examined; hence, 1022 completed the examination. An additional 72 participants did not complete HRV measurements because of an unstable heart rate (HR). Finally, 950 participants were included in the analysis.

Table 1 shows the clinical characteristics of the study participants. The characteristics of the study cohort were as follows: sex distribution, 58.9% women and 41.1% men; mean age, 52.6 years; and mean body mass index (BMI), 23.1 kg/m^2^. In the HRV parameters, the mean intra-individual coefficient of variation of the RR interval (CVRR) and the inter-individual CVRR were 3.4% and 14.0%, respectively. Table 2 shows the relative proportions of the main gut microbial phyla among participants. The mean proportions were as follows: Actinobacteria, 11.9%; Bacteroidetes, 24.8%; Proteobacteria, 2.9%; and Firmicutes, 58.9%. For reference, the summary statistics of 52 gut microbial genera possessed by more than 50% of the participants are shown in Supplementary Materials Table S1.

Table 3 and Table 4 show associations between HRV parameters and diversity (Simpson and Shannon indices) of the gut microbial genus. A higher Simpson index was associated with a higher standard deviation of RR intervals (SDNN) in multivariate regression analysis of Model 2 (β = 0.213; 95% confidence interval [CI], 0.012 to 0.413; *p* = 0.038). A higher Shannon index was associated with a higher SDNN in the multivariate regression analysis of Model 2 (β = 3.934; 95% CI, 0.444 to 7.424; *p* = 0.027). 

These results showed that a higher gut microbial diversity was associated with a higher SDNN after adjusting for age, sex, BMI, antidiabetic, antihyperlipidemic, antihypertensive, physical activity, smoking, and alcohol consumption. However, no significant associations with CVRR, low-frequency component power (LF), high-frequency component power (HF), LF/HF, and HR were confirmed.

Table 5 shows the associations between HRV parameters and *Lachnospiraceae incertae sedis*. In the univariate and multivariate regression analyses, *L. incertae sedis* was the only gut microbiota associated with SDNN (Tables S2–S4). Multivariate regression analysis (Model 2) revealed that a higher relative count of *L. incertae sedis* was associated with a higher SDNN (β = 1.449; 95% CI, 0.616 to 2.282; *p* = 0.001), CVRR (β = 0.135; 95% CI, 0.045 to 0.225; *p* = 0.003), LF (β = 59.687; 95% CI, 28.954 to 90.420; *p* < 0.001), and LF/HF (β = 0.367; 95% CI, 0.124 to 0.609; *p* = 0.003). In addition, HR was associated with a higher relative count of *L. incertae sedis* in the multivariate regression analysis of Model 1 only (β = −0.626; 95% CI, −1.212 to −0.040; *p* = 0.036).

These results indicated that higher levels of HRV parameters, such as SDNN, CVRR, LF, and LF/HF, were associated with a higher relative count of *L. incertae sedis* after adjusting for age, sex, BMI, antidiabetic, antihyperlipidemic, antihypertensive, physical activity, smoking, and alcohol consumption.

## 3. Discussion

Among the participants in the Iwaki Health Promotion Project medical examination, a higher gut microbial diversity and *L. incertae sedis* abundance were associated with higher HRV parameters. In this study, the relative counts of the four major phyla in the gut (Firmicutes, Bacteroidetes, Actinobacteria, and Proteobacteria) were similar to those reported among participants in urban Kyoto City [13]. This study investigated the gut microbiota in the general Japanese population. A decreased gut microbial diversity has been linked to inflammatory bowel disease (IBD) [14], irritable bowel syndrome (IBS) [15], obesity [16], and a Western diet high in fat and sugar compared to a low-fat plant-based diet [17]. Understanding gut microbial diversity could facilitate the development of personalized nutritional and drug strategies [18].

*L. incertae sedis* abundance was associated with higher HRV parameters such as SDNN, CVRR, LF, and LF/HF, suggesting its association with overall cardiac autonomic and sympathetic activity. These findings are relevant because, to the best of our knowledge, no other study has reported an association between HRV and gut microbiota abundance among many participants. *L*. *incertae sedis* belongs to the *Lachnospiraceae* family phylogenetically and morphologically heterogeneous taxon belonging to the clostridial cluster XIVa of the phylum Firmicutes, which hydrolyzes starch and other sugars to produce butyrate and other short-chain fatty acids (SCFAs) [19]. The clostridial cluster XIVa, derived from human feces, may induce T regulatory cells and suppress inflammatory conditions such as IBD via SCFA butyrate production [20,21]. Previous studies have reported a higher prevalence of clostridial cluster XIVa in rural Kyotango City than in the urban city of Kyoto [13]. Kyotango City is a long-lived province with multiple centenarians, and emerging evidence suggests that the brain–gut interaction may influence the etiology of IBD. Clinical studies have shown that alterations in the brain–gut interaction are associated with autonomic nervous system dysfunction, affecting the central nervous and digestive systems [22]. Crohn’s disease (CD) and ulcerative colitis (UC) are classified as IBD as they have similar symptoms and lead to digestive disorders and inflammation in the digestive system [23]. Patients with UC and CD have lower time-domain parameters, such as SDNN, and HRV frequency domain parameters, such as LF and HF, than healthy participants [22]. The relationship between *L. incertae sedis* contained in butyrate-producing bacterium and some HRV parameters was confirmed in this study; hence, further studies to investigate the relationship between butyrate-producing bacteria and IBD via HRV are warranted.

Moreover, butyrate-producing bacteria reduce obesity by modulating G-protein coupled receptors (GPRs) 41 and 43 [24,25]. GPR-41 is most abundantly expressed in sympathetic ganglia in mice and humans, indicating its importance in these cells [24]. Obesity develops when energy intake exceeds energy expenditure; thus, increasing cellular energy expenditure may be an attractive approach [26]. Direct regulation of sympathetic activation via GPR-41 may serve as a key physiological mechanism regulating the body’s energy balance since modulating sympathetic nervous system (SNS) activity causes an increase or decrease in energy expenditure [24]. Incidentally, it has been reported that obesity lowers SDNN; however, no association between obesity and HRV parameters related to SNS was identified [27,28]. Whether targeting the SNS directly improves obesity or metabolism remains unknown but merits further attention [29]. In obesity, the composition of the intestinal flora is disrupted and is associated with cardiac and HRV dysfunction [30]. This study results highlight the importance of further investigating the anti-obesity and cardioprotective effects of prebiotics, probiotics, and synbiotics of butyrate-producing bacteria.

Changes in the gut microbiota composition and gut microbial metabolism have been implicated in the etiology of CVD. Gut-derived metabolites play important roles in maintaining healthy cardiac and vascular function [9,10,11,12]. CVD, as with IBD, is characterized by chronic inflammation and exhibits similar physiological mechanisms [31]. Furthermore, gastrointestinal disorders generally occurs such as IBS when the autonomic nervous system fails to regulate gastrointestinal motility [32]. Previous studies have suggested a relationship between IBS and the gut microbiota [15]. Clinical applications of the relationship between autonomic nerves and the gut microbiota may contribute to the prevention and treatment of CVD, IBD, and IBS.

In addition, the association between the gut and vaginal microbiota has recently attracted attention. The vaginal microbiota, as with the gut microbiota, have been reported to be associated with HRV and suggested to be related to an anti-inflammatory response due to autonomic nervous activity during parturition [33]. The relationship between the gut and vaginal microbiota is thought to be due to the vagina’s proximity to the anus, which allows the gut microbiota to reach the reproductive organs via the rectum and perineum [33]. Further studies on the relationship between the gut microbiota and the vaginal microbiota via autonomic nervous system are warranted.

This study had some limitations. First, the measurement of HRV was not optimal. Generally, HRV should be measured in the supine position for at least 24 h or 5 min [34]. However, in this study, measurements were taken in the sitting position for a relatively short time, 90 s [35]. Therefore, individuals with unstable HR and those who did not measure HRV were excluded from the analysis, which may have biased the results [35]. Furthermore, only limited HRV parameters were analyzed in this study; the association between HRV parameters not measured in VM302 and the gut microbiota could not be determined. In addition, methods that analyze HRV in the time or frequency domain may not be sufficient to characterize the complex dynamics of the heartbeat [36]. More reliable non-linear regression analyses are required to calculate HRV parameters. Second, although we adjusted for antidiabetic, antihyperlipidemic and antihypertensive use, the effects of specific classes of these medications that may be related to cardiac autonomic function and the gut microbiota could not be completely surveyed. Third, because of the cross-sectional design [34], we could not determine a causal relationship between HRV and gut microbiota abundance. Longitudinal data are needed to elucidate this relationship. Fourth, this study included participants who voluntarily participated in the Iwaki Health Promotion Health Examination [34]. Therefore, we may not be able to generalize the results of this study due to selection bias [34]; additional longitudinal studies with large sample sizes are needed to clarify the causal relationship between HRV and gut microbiota abundance.

## 4. Materials and Methods

### 4.1. Participants and Analysis

The Iwaki Health Promotion Project Health Examination has been conducted annually since 2005 to prevent lifestyle-related diseases, maintain and promote health, and increase longevity among residents of the Iwaki area of Hirosaki City [37]. The participants (*n* = 1065) in the 2019 health examination were men and women aged 20 years or older living in the Iwaki area of Hirosaki City, Aomori Prefecture [34]. This cross-sectional study included 950 participants who completed HRV and gut microbiota measurements. This study was approved by the Hirosaki University School of Medicine Ethics Review Committee (approval number: 2019-009) and was conducted in accordance with the principles of the Declaration of Helsinki. Written informed consent was obtained from all participants [37].

### 4.2. Clinical Features

All clinical examinations were performed in the morning in a fasting state [34]. BMI was assessed by physical examination, and systolic and diastolic blood pressures were measured. BMI was calculated from body weight and height (kg/m^2^). Blood pressure was measured with an automatic blood pressure meter Elemano 2 (Terumo Corporation, Tokyo, Japan) while the participant was seated and at rest. Blood tests were performed to determine glucose metabolic capacity by measuring hemoglobin A1c, glycoalbumin, and blood glucose; lipid metabolic capacity by measuring triglycerides, total cholesterol, high-density lipoprotein, and low-density lipoprotein; and liver function by measuring alanine transaminase, aspartate transaminase, γ-glutamyl transaminases were measured to evaluate liver function, and creatinine and urea nitrogen were measured to evaluate kidney function. Blood samples were collected from peripheral veins in the supine position, and blood tests were performed at LSI Medience Company (Tokyo, Japan) [34]. For analysis of lifestyle-related factors, investigations were collected for diabetes, dyslipidemia, hypertension, heart disease, gastric/duodenal ulcer, smoking, alcohol consumption, exercise (non-winter and winter), antidiabetic, antihyperlipidemic, and antihypertensive.

### 4.3. Measurement of HRV

HRV was measured in the morning while fasting [34]. Participants did not smoke or engage in strenuous exercise during the physical examination [34]. A Vital Monitor 302 (VM302) system (Hitachi Systems, Ltd., Tokyo, Japan) was used to ensure that the HR was stable, and measurements were taken for 90 s with the participant seated and eyes closed [34]. Data were analyzed using Memfmcc software (Fatigue Science Institute, Osaka, Japan) [34]. The VM302 can simultaneously perform electrocardiography (ECG) and photoelectric volumetric pulse waves from the fingertip and has been used in several clinical trials [38,39]. By monitoring HRV with ECG and photoplethysmography, 90 s of cardiac autonomic function data were collected at a sampling rate of 600 Hz [34]. The VM302′s built-in firmware uses a peak detection algorithm based on the Hill climbing method to detect the R-wave peak and transmits the obtained R-wave peak times to an external computer [34]. A time series of RR intervals was sequentially generated from the peak time of each R wave [34]. Noise in the digital signal was removed with a low-pass filter, but not the linear trend of the RR interval. The HR was calculated from the reciprocal of the RR interval for each heartbeat. In addition, instead of a steady-state test for RR interval variability, the error of the data for 30 s of the RR interval was set at 0.75-fold lower and 1.75-fold higher than the median value of the RR interval for 30 s, and the matched RR interval data were removed [34].

SDNN and CVRR, which express modulation of sympathetic and parasympathetic functions as time-domain parameters, were evaluated [34,40]. SDNN and CVRR were calculated as the SDNN and SDNN/mean value of the RR interval × 100, during the measurement. CVRR is SDNN normalized to the RR interval [41]. Variations in the RR interval are typical HRV parameters [42]. In addition, parameters in the frequency domain were evaluated; LF was calculated as power in the frequency range of 0.04 to 0.15 Hz, and HF as power in the frequency range of 0.15 to 0.4 Hz. The average values of LF, HF, and LF/HF obtained for each time series were representative of each measurement [34]. Frequency analysis of RR interval variation was performed using the maximum entropy method, which allows the estimation of power spectral density from short time series data and is suitable for studying HRV changes under different conditions over short periods [34].

### 4.4. Measurements of the Gut Microbiota

The detailed processing method for extracting deoxyribonucleic acid (DNA) from fecal samples is shown below [43]. Each participant promptly transferred 2–3 g of fresh feces into a storage container (TechnoSuruga Laboratory, Shizuoka, Japan) containing 3 mL of a guanidine thiocyanate stock solution (GTC buffer; 100 mM Tris-HCl [pH 9.0], 40 mM Tris-EDTA [pH 8.0], and 4 M guanidine thiocyanate). Fecal samples were stored at room temperature. Fecal sample suspensions (200 μL) were added to a tube with zirconium beads and 800 μL of GTC buffer and homogenized using a FastPrep 24 Instrument (MP Biomedicals, Santa Ana, CA, USA) at 5 m/s for 2 min. After cooling, the samples were centrifuged at 2400× *g* for 1 min. DNA was then purified from the bead-treated suspension using an automated DNA isolation system (GENE PREP STAR PI-480, KURABO, Osaka, Japan).

The detailed processing method for analyzing the gut microbiota using next-generation sequencing is mentioned here [44]. Sequences of the V3–V4 region of 16S recombinant DNA were used to identify the bacteria. Amplified fragments were purified using polymerase chain reaction (PCR) cleanup filter plates (Merck Millipore, Burlington, MA, USA) and quantified by real-time quantitative PCR. Bacterial sequences were detected and identified using the Illumina MiSeq system (Illumina, San Diego, CA, USA).

The detailed processing method for multiplexed paired-end reads is shown below [45]. Adaptor sequences and low-quality bases (threshold = 20) were trimmed at the 3′-end of the reads by Cutadapt (version: 1.13) [46]. N bases and reads containing less than 150 bases containing reads were discarded. Paired-end reads that exceeded the filter threshold were merged into a single read called a “merged read.” Merged reads < 370 or >470 bases were excluded by the fastq_mergepairs subcommand of VSEARCH (version: 2.4.3) [47]. Merged reads with multiple expected sequence errors were also excluded. After removing chimera reads detected by the uchime denovo subcommand of VSEARCH, the remaining merged reads were clustered with a sequence identity ≥ 97%. Taxonomy of the identified clusters was predicted by applying ribosomal database project Classifier (commit hash: 701e229dde7cbe53d4261301e23459d91615999d) based on representative reads. Results with confidence values less than 0.8 were treated as unclassified. The composition ratio of each genus of the gut microbiota was determined by dividing the number of read counts of each genus by the total number of read counts.

Of the 294 genera detected (including unclassified), genera not possessed by more than 50% of the participants, which were too few to analyze accurately, were excluded from the analysis.

### 4.5. Statistical Analysis

Among the clinical characteristics of the participants, continuous variables are presented as the means and standard deviations, whereas categorical variables are presented as the frequency and percentages. The relative counts of gut microbial phylum and genera are presented as the means and standard deviations. To investigate the diversity (alpha-diversity) of microbial genus level associated with HRV, univariate and multivariate regression analyses were performed with various HRV parameters as the objective variables and the Simpson or Shannon index [48] as the explanatory variables. To investigate the relative counts of gut microbial genera associated with HRV, univariate and multivariate regression analyses were performed with the specific HRV parameter related to microbial diversity as the objective variables and the abundances of each gut microbial genus as the explanatory variables. Moreover, univariate and multivariate regression analyses were performed with various HRV parameters as the objective variables and abundances of the specific gut microbial genus detected above as the explanatory variables. In the multivariate analysis, Model 1 (adjusted for age, sex, and BMI) and Model 2 (adjusted for antidiabetic, antihyperlipidemic, antihypertensive, physical activity, smoking, and alcohol consumption in addition to age, sex, and BMI) were performed. The regression coefficient (β) was calculated using the standard least squares method. In these analyses, the significance level was set at *p* < 0.05 in two-tailed tests, and 95% CI for the regression coefficient (β) was calculated. We used JMP^®^ Ver.16 software (SAS Institute, Inc., Cary, NC, USA) in these statistical analyses [34].

## 5. Conclusions

This study showed that a higher gut microbial diversity was associated with a higher SDNN. Moreover, *L. incertae sedis* abundance was associated with higher HRV parameters such as SDNN, CVRR, LF, and LF/HF. In the general Japanese population, higher gut microbial diversity and *L. incertae sedis* abundance were associated with higher HRV parameters. Clinical applications of autonomic nerves and the gut microbiota possibly contribute to the prevention and treatment of IBS, IBD, and CVD.

## Figures and Tables

**Table 1:** Participant characteristics.

Continuous Variables		Total (*N* = 950)		Men (*N* = 391)		Women (*N* = 559)	
	(Unit)	Mean	(SD)	Mean	(SD)	Mean	(SD)
Age	(years)	52.6	(14.77)	52.4	(14.58)	52.7	(14.90)
BMI	(kg/m^2^)	23.1	(3.63)	24.1	(3.51)	22.4	(3.54)
HbA1c	(%)	5.7	(0.62)	5.7	(0.71)	5.7	(0.54)
Glycoalbumin	(%)	14.6	(1.98)	14.4	(2.41)	14.7	(1.61)
Blood glucose	(mg/dL)	96.3	(16.52)	99.8	(18.29)	93.9	(14.70)
Triglyceride	(mg/dL)	98.2	(84.33)	125.2	(114.54)	79.4	(45.43)
Total cholesterol	(mg/dL)	205.4	(34.27)	203.0	(34.17)	207.0	(34.27)
HDL cholesterol	(mg/dL)	65.0	(16.66)	58.2	(15.00)	69.8	(16.08)
LDL cholesterol	(mg/dL)	116.8	(29.79)	117.4	(29.34)	116.4	(30.11)
ALT	(U/L)	20.9	(13.75)	26.5	(16.52)	17.0	(9.69)
AST	(U/L)	21.8	(7.86)	23.8	(8.37)	20.5	(7.18)
γ-GTP	(U/L)	33.2	(40.94)	49.1	(56.98)	22.1	(16.75)
Creatinine	(mg/dL)	0.7	(0.55)	0.9	(0.74)	0.6	(0.33)
Urea nitrogen	(mg/dL)	14.5	(4.50)	15.4	(4.52)	14.0	(4.40)
SBP	(mmHg)	120.8	(16.88)	123.5	(16.65)	118.9	(16.79)
DBP	(mmHg)	77.1	(11.20)	79.5	(11.48)	75.3	(10.67)
RR interval	(ms)	866.9	(121.76)	885.9	(127.19)	853.7	(116.09)
CVRR	(%)	3.4	(1.67)	3.4	(1.70)	3.4	(1.56)
SDNN	(ms)	29.3	(14.56)	29.8	(15.7)	29.0	(13.76)
LF	(ms^2^)	343.5	(522.26)	421.4	(625.19)	289.0	(428.32)
HF	(ms^2^)	252.2	(340.90)	246.9	(364.04)	256.0	(324.03)
LF/HF	(Ratio)	2.7	(3.99)	3.3	(4.73)	2.2	(3.32)
HR	(bpm)	70.1	(10.15)	68.7	(10.23)	71.1	(9.98)
**Categorical variables**		***N***	**(%)**	***N***	**(%)**	***N***	**(%)**
Diabetes mellitus	No	890	(93.9%)	361	(92.3%)	529	(95.0%)
	Yes	58	(6.1%)	30	(7.7%)	28	(5.0%)
Hyperlipidemia	No	778	(82.2%)	315	(81.0%)	463	(83.1%)
	Yes	168	(17.8%)	74	(19.0%)	94	(16.9%)
High blood pressure	No	711	(74.9%)	275	(70.3%)	436	(78.1%)
	Yes	238	(25.1%)	116	(29.7%)	122	(21.9%)
Heart disease	No	906	(95.5%)	366	(93.6%)	540	(96.8%)
	Yes	43	(4.5%)	25	(6.4%)	18	(3.2%)
Gastric/Duodenal ulcer	No	853	(89.9%)	342	(87.5%)	511	(91.6%)
	Yes	96	(10.1%)	49	(12.5%)	47	(8.4%)
Antidiabetic medication use	No	903	(95.1%)	367	(93.9%)	536	(95.9%)
	Yes	47	(4.9%)	24	(6.1%)	23	(4.1%)
Antihyperlipidemic medication use	No	846	(89.1%)	346	(88.5%)	500	(89.4%)
	Yes	104	(10.9%)	45	(11.5%)	59	(10.6%)
Antihypertensive medication use	No	728	(76.6%)	285	(72.9%)	443	(79.2%)
	Yes	222	(23.4%)	106	(27.1%)	116	(20.8%)
Exercising (non-winter)	No	732	(77.4%)	302	(77.6%)	430	(77.2%)
	Yes	214	(22.6%)	87	(22.4%)	127	(22.8%)
Exercising (winter)	No	731	(77.7%)	304	(78.6%)	427	(77.1%)
	Yes	210	(22.3%)	83	(21.4%)	127	(22.9%)
Smoking	No	596	(63.3%)	161	(41.6%)	435	(78.4%)
	Current	161	(17.1%)	114	(29.5%)	47	(8.5%)
	Previous	185	(19.6%)	112	(28.9%)	73	(13.2%)
Alcohol consumption	No	444	(47.3%)	111	(28.6%)	333	(60.5%)
	Current	456	(48.6%)	266	(68.6%)	190	(34.5%)
	Previous	38	(4.1%)	11	(2.8%)	27	(4.9%)

Abbreviations: γ-GTP, γ-glutamyl transferase; ALT, alanine transaminase; AST, aspartate transaminase; BMI, body mass index; bpm, beats per minute; CVRR, coefficient of variation of RR intervals; DBP, diastolic blood pressure; HbA1c, hemoglobin A1c; HDL, high-density lipoprotein; HF, high-frequency component power; HR, heart rate; LDL, low-density lipoprotein; LF, low-frequency component power; SBP, systolic blood pressure; SD, standard deviation; SDNN, standard deviation of RR intervals.

**Table 2:** Relative count of gut microbial major phylum in participant.

Continuous Variables		Total (*N* = 950)		Men (*N* = 391)		Women (*N* = 559)	
	(Unit)	Mean	(SD)	Mean	(SD)	Mean	(SD)
Actinobacteria	(%)	11.9	(9.02)	11.8	(9.31)	12.0	(8.82)
Bacteroidetes	(%)	24.8	(11.22)	26.7	(12.47)	23.5	(10.05)
Proteobacteria	(%)	2.9	(2.56)	3.2	(2.76)	2.6	(2.38)
Firmicutes	(%)	58.9	(12.27)	56.2	(13.04)	60.8	(11.33)

Abbreviation: SD, standard deviation.

**Table 3:** Analysis of the association between HRV parameters and the Simpson index (%).

Characteristics	Univariate			Model 1			Model 2		
	β	95% CI	*p*-Value	β	95% CI	*p*-Value	β	95% CI	*p*-Value
SDNN (ms)	0.113	−0.093 to 0.320	0.282	0.174	−0.020 to 0.369	0.078	0.213	0.012 to 0.413	0.038
CVRR (%)	0.011	−0.012 to 0.034	0.368	0.017	−0.004 to 0.038	0.116	0.020	−0.002 to 0.042	0.070
LF (ms^2^)	0.743	−6.673 to 8.159	0.844	3.963	−3.174 to 11.100	0.276	4.598	−2.823 to 12.018	0.224
HF (ms^2^)	0.564	−4.277 to 5.405	0.819	1.024	−3.738 to 5.786	0.673	2.305	−2.589 to 7.199	0.356
LF/HF	0.013	−0.043 to 0.070	0.641	0.034	−0.022 to 0.090	0.236	0.034	−0.024 to 0.092	0.254
HR (bpm)	−0.022	−0.166 to 0.122	0.762	−0.033	−0.175 to 0.109	0.648	−0.051	−0.195 to 0.093	0.484

Model 1: Adjusted for age, sex, and BMI. Model 2: Adjusted for age, sex, BMI, antidiabetic, antihyperlipidemic, antihypertensive, physical activity (non-winter and winter months), smoking, and alcohol consumption. Abbreviations: BMI, body mass index; CI, confidence interval; CVRR, coefficient of variation of RR intervals; HF, high-frequency component power; HR, heart rate; HRV, heart rate variability; LF, low-frequency component power; SDNN, standard deviation of RR intervals.

**Table 4:** Analysis of the association between HRV parameters and the Shannon index.

Characteristics	Univariate			Model 1			Model 2		
	β	95% CI	*p*-Value	β	95% CI	*p*-Value	β	95% CI	*p*-Value
SDNN (ms)	0.681	−2.931 to 4.293	0.711	3.542	0.142 to 6.942	0.041	3.934	0.444 to 7.424	0.027
CVRR (%)	−0.056	−0.458 to 0.346	0.786	0.302	−0.064 to 0.667	0.106	0.346	−0.030 to 0.721	0.071
LF (ms^2^)	−38.512	−168.009 to 90.984	0.560	57.731	−67.340 to 182.803	0.365	63.993	−65.218 to 193.205	0.331
HF (ms^2^)	3.120	−81.242 to 87.663	0.942	38.975	−44.438 to 122.388	0.359	50.440	−34.732 to 135.612	0.245
LF/HF	−0.014	−1.003 to 0.975	0.978	0.342	−0.647 to 1.331	0.498	0.346	−0.671 to 1.363	0.504
HR (bpm)	−2.232	−4.745 to 0.280	0.082	−1.608	−4.088 to 0.872	0.204	0.676	−4.132 to 0.876	0.202

Model 1: Adjusted for age, sex, and BMI. Model 2: Adjusted for age, sex, BMI, antidiabetic, antihyperlipidemic, antihypertensive, physical activity (non-winter and winter months), smoking, and alcohol consumption. Abbreviations: BMI, body mass index; CI, confidence interval; CVRR, coefficient of variation of RR intervals; HF, high-frequency component power; HR, heart rate; HRV, heart rate variability; LF, low-frequency component power; SDNN, standard deviation of RR intervals.

**Table 5:** Analysis of the association between HRV parameters and the relative count (%) of *Lachnospiraceae* incertae *sedis*.

Characteristics	Univariate			Model 1			Model 2		
	β	95% CI	*p*-Value	β	95% CI	*p*-Value	β	95% CI	*p*-Value
SDNN (ms)	1.024	0.169 to 1.878	0.019	1.390	0.589 to 2.191	0.001	1.449	0.616 to 2.282	0.001
CVRR (%)	0.087	−0.008 to 0.183	0.072	0.127	0.040 to 0.213	0.004	0.135	0.045 to 0.225	0.003
LF (ms^2^)	41.007	10.402 to 71.611	0.009	57.949	28.590 to 87.309	<0.001	59.687	28.954 to 90.420	<0.001
HF (ms^2^)	7.118	−12.926 to 27.163	0.486	10.824	−8.899 to 30.548	0.282	11.525	−8.885 to 31.935	0.268
LF/HF	0.271	0.037 to 0.505	0.023	0.357	0.124 to 0.590	0.003	0.367	0.124 to 0.609	0.003
HR (bpm)	−0.586	−1.182 to 0.010	0.054	−0.626	−1.212 to −0.040	0.036	−0.576	−1.175 to 0.023	0.060

Model 1: Adjusted for age, sex, and BMI. Model 2: Adjusted for age, sex, BMI, antidiabetic, antihyperlipidemic, antihypertensive, physical activity (non-winter and winter months), smoking, and alcohol consumption. Abbreviations: BMI, body mass index; CI, confidence interval; CVRR, coefficient of variation of RR intervals; HF, high-frequency component power; HR, heart rate; HRV, heart rate variability; LF, low-frequency component power; SDNN, standard deviation of RR intervals.

## Data Availability

The data cannot be shared publicly because of ethical concerns. Data are available from the Hirosaki University COI Program Institutional Data Access/Ethics Committee (contact via e-mail: coi@hirosaki-u.ac.jp) for researchers who meet the criteria for access to the data. Researchers need to have prior approval from the research ethics review boards of their respective affiliations.

## Supplementary Materials

The following supporting information can be downloaded at: https://www.mdpi.com/article/10.3390/metabo12080730/s1, Table S1: The summary statistics of the relative count (%) of gut microbial genera. Table S2: Univariate analysis of the association between SDNN (ms) and the relative count (%) of gut microbial genera. Table S3: Multivariate analysis (Model 1) of the association between SDNN (ms) and the relative count (%) of gut microbial genera. Table S4: Multivariate analysis (Model 2) of the association between SDNN (ms) and the relative count (%) of gut microbial genera.
