# Supplementary material for: Association of Gut Microbial Genera with Heart Rate Variability in the General Japanese Population: The Iwaki Cross-Sectional Research Study
Source: Metabolites. 2022 Aug 7;12(8):730. doi: 10.3390/metabo12080730 (PMC9414323; doi:10.3390/metabo12080730)
Supplement: Supplementary file 1 [file metabolites-12-00730-s001.zip › metabolites-1808987-supplementary.pdf]

Table S1. The summary statistics of the relative count (%) of gut microbial genera.

Gut microbial genera	Total (*N* = 950)	Total (*N* = 950)		Men (*N* = 391)		Women (*N* = 559)	
	***N* of detected**	**Mean**	**(SD)**	**Mean**	**(SD)**	**Mean**	**(SD)**
Akkermansia	479	0.580	(2.310)	0.535	(2.931)	0.611	(1.751)
Fusobacterium	490	0.626	(2.644)	1.171	(3.827)	0.245	(1.137)
Megasphaera	502	0.404	(1.114)	0.571	(1.352)	0.288	(0.893)
Anaerotruncus	506	0.016	(0.033)	0.011	(0.027)	0.020	(0.037)
Saccharibacteria_genera_incertae_sedis	550	0.026	(0.052)	0.031	(0.070)	0.022	(0.034)
Holdemania	553	0.016	(0.026)	0.015	(0.027)	0.017	(0.025)
Bacillus	560	0.082	(0.235)	0.073	(0.191)	0.089	(0.262)
Intestinimonas	563	0.053	(0.103)	0.031	(0.064)	0.068	(0.121)
Dialister	567	0.286	(0.547)	0.265	(0.595)	0.301	(0.510)
Bilophila	608	0.165	(0.238)	0.146	(0.213)	0.177	(0.254)
Granulicatella	627	0.005	(0.008)	0.005	(0.008)	0.004	(0.008)
Haemophilus	640	0.091	(0.502)	0.080	(0.256)	0.098	(0.619)
Sutterella	642	1.135	(1.672)	1.579	(1.959)	0.824	(1.355)
Parasutterella	644	0.559	(1.093)	0.428	(0.924)	0.650	(1.189)
Odoribacter	655	0.133	(0.233)	0.102	(0.168)	0.154	(0.267)
Phascolarctobacterium	688	0.628	(0.863)	0.852	(0.956)	0.471	(0.754)
Turicibacter	688	0.280	(0.929)	0.289	(1.093)	0.273	(0.795)
Lactobacillus	691	0.341	(1.759)	0.476	(2.214)	0.247	(1.347)
Coprococcus	719	0.499	(0.764)	0.460	(0.621)	0.527	(0.849)
Intestinibacter	738	0.136	(0.291)	0.112	(0.281)	0.152	(0.297)
Clostridium sensu stricto	741	0.236	(1.332)	0.267	(1.429)	0.214	(1.260)
Escherichia/Shigella	775	0.346	(1.393)	0.401	(1.731)	0.307	(1.097)
Prevotella	798	6.414	(11.970)	9.581	(13.964)	4.200	(9.770)
Erysipelotrichaceae_incertae_sedis	802	0.426	(1.244)	0.414	(1.418)	0.434	(1.108)
Ruminococcus	812	3.125	(4.177)	2.253	(3.727)	3.735	(4.365)
Romboutsia	825	0.317	(0.655)	0.312	(0.709)	0.321	(0.616)
Collinsella	839	3.943	(4.060)	4.599	(4.143)	3.483	(3.940)
Veillonella	842	0.668	(1.537)	0.598	(1.680)	0.717	(1.429)
Alistipes	848	2.490	(3.542)	1.633	(2.778)	3.090	(3.882)
Eggerthella	849	0.171	(0.257)	0.122	(0.193)	0.206	(0.288)
Actinomyces	861	0.041	(0.058)	0.045	(0.053)	0.038	(0.061)
Gemmiger	867	2.736	(2.709)	2.325	(2.510)	3.024	(2.806)
Fusicatenibacter	868	2.021	(2.020)	1.919	(2.013)	2.093	(2.023)

Dorea	887	0.814	(0.753)	0.902	(0.778)	0.752	(0.729)
Oscillibacter	895	0.406	(0.513)	0.301	(0.407)	0.479	(0.564)
Clostridium XlVb	899	0.199	(0.311)	0.200	(0.325)	0.198	(0.301)
Parabacteroides	916	1.126	(1.705)	1.004	(1.111)	1.211	(2.017)
Clostridium XVIII	920	0.530	(0.874)	0.572	(1.038)	0.500	(0.738)
Roseburia	922	3.846	(3.876)	3.686	(3.785)	3.957	(3.939)
Flavonifractor	930	0.232	(0.271)	0.184	(0.244)	0.265	(0.283)
Ruminococcus2	931	4.860	(5.211)	4.845	(5.516)	4.870	(4.992)
Butyricicoccus	936	0.742	(0.563)	0.802	(0.560)	0.701	(0.561)
Clostridium IV	942	2.657	(4.214)	2.058	(3.382)	3.075	(4.667)
Faecalibacterium	943	7.523	(5.539)	6.641	(5.311)	8.139	(5.616)
Anaerostipes	944	4.574	(4.856)	4.655	(5.117)	4.516	(4.669)
Bifidobacterium	944	7.172	(7.558)	6.431	(7.593)	7.691	(7.498)
Clostridium XlVa	946	0.381	(0.332)	0.339	(0.284)	0.411	(0.359)
Streptococcus	949	1.525	(3.006)	1.533	(3.177)	1.520	(2.882)
Bacteroides	950	13.489	(8.829)	12.873	(9.252)	13.920	(8.503)
Blautia	950	7.093	(4.176)	6.832	(3.839)	7.275	(4.390)
Lachnospiracea_incertae_sedis	950	1.609	(1.084)	1.441	(0.951)	1.727	(1.154)
Unclassified	950	8.195	(4.820)	7.783	(5.073)	8.484	(4.618)

Abbreviations: SD, standard deviation

Table S2. Univariate analysis of the association between SDNN (ms) and the relative count (%) of gut microbial genera.

Gut microbial genera	Univariate				
	**β**	**95% CI**			***p*-value**
Intestinibacter	-4.217	-7.393	to	-1.041	0.009
Blautia	0.273	0.051	to	0.495	0.016
Lachnospiracea_incertae_sedis	1.024	0.169	to	1.878	0.019
Lactobacillus	-0.622	-1.148	to	-0.096	0.021
Bilophila	-4.430	-8.317	to	-0.543	0.026
Anaerostipes	0.199	0.008	to	0.389	0.041
Eggerthella	3.206	-0.404	to	6.817	0.082
Flavonifractor	3.013	-0.410	to	6.436	0.084
Clostridium IV	0.186	-0.034	to	0.406	0.097
Parasutterella	0.715	-0.133	to	1.563	0.098
Bacillus	-3.189	-7.125	to	0.748	0.112

Clostridium sensu stricto	-0.547	-1.243	to	0.149	0.123
Odoribacter	2.749	-1.234	to	6.732	0.176
Unclassified	-0.131	-0.323	to	0.061	0.182
Saccharibacteria_genera_incertae_sedis	-11.557	-29.363	to	6.250	0.203
Akkermansia	-0.259	-0.660	to	0.143	0.206
Turicibacter	-0.638	-1.637	to	0.360	0.210
Dorea	0.767	-0.465	to	1.999	0.222
Phascolarctobacterium	-0.647	-1.722	to	0.428	0.238
Granulicatella	-67.584	-183.417	to	48.250	0.252
Haemophilus	-1.021	-2.868	to	0.825	0.278
Butyricicoccus	-0.828	-2.478	to	0.821	0.324
Prevotella	-0.038	-0.115	to	0.040	0.340
Actinomyces	-7.546	-23.543	to	8.451	0.355
Clostridium XlVa	-1.303	-4.098	to	1.493	0.361
Escherichia/Shigella	-0.308	-0.974	to	0.358	0.365
Streptococcus	-0.138	-0.447	to	0.170	0.379
Clostridium XVIII	-0.443	-1.504	to	0.619	0.413
Anaerotruncus	-10.038	-37.746	to	17.669	0.477
Dialister	0.536	-1.162	to	2.233	0.536
Roseburia	-0.063	-0.302	to	0.177	0.608
Bifidobacterium	0.030	-0.092	to	0.153	0.626
Erysipelotrichaceae_incertae_sedis	-0.173	-0.919	to	0.573	0.650
Romboutsia	-0.319	-1.736	to	1.097	0.658
Megasphaera	0.162	-0.672	to	0.995	0.703
Intestinimonas	1.636	-7.374	to	10.645	0.722
Sutterella	-0.096	-0.651	to	0.459	0.735
Alistipes	-0.045	-0.307	to	0.217	0.738
Ruminococcus	-0.035	-0.257	to	0.188	0.759
Veillonella	0.084	-0.520	to	0.688	0.784
Gemmiger	0.037	-0.305	to	0.380	0.831
Oscillibacter	-0.172	-1.983	to	1.638	0.852
Parabacteroides	-0.049	-0.593	to	0.496	0.861
Collinsella	0.017	-0.212	to	0.245	0.887
Holdemania	2.573	-33.352	to	38.498	0.888
Clostridium XlVb	-0.206	-3.188	to	2.775	0.892
Bacteroides	0.007	-0.098	to	0.112	0.901

Fusobacterium	-0.020	-0.371	to	0.331	0.912
Fusicatenibacter	-0.011	-0.471	to	0.448	0.961
Faecalibacterium	-0.003	-0.170	to	0.165	0.974
Ruminococcus2	0.002	-0.176	to	0.180	0.982
Coprococcus	-0.014	-1.229	to	1.201	0.982

Abbreviations: CI, confidence interval; SDNN, standard deviation of RR intervals.

Table S3. Multivariate analysis (model 1) of the association between SDNN (ms) and the relative count (%) of gut microbial genera.

**Gut microbial genera**	**Model 1**				
	**β**	**95% CI**			***p*-value**
Lachnospiracea_incertae_sedis	1.390	0.589	to	2.191	0.001
Bilophila	-3.254	-6.891	to	0.384	0.080
Romboutsia	1.127	-0.213	to	2.467	0.099
Clostridium IV	0.172	-0.034	to	0.379	0.101
Granulicatella	-88.871	-196.795	to	19.054	0.106
Bacteroides	-0.080	-0.179	to	0.019	0.115
Erysipelotrichaceae_incertae_sedis	-0.501	-1.197	to	0.195	0.116
Ruminococcus	0.150	-0.062	to	0.362	0.158
Dorea	0.752	-0.401	to	1.906	0.166
Saccharibacteria_genera_incertae_sedis	-10.641	-27.289	to	6.008	0.201
Clostridium XlVa	-1.577	-4.195	to	1.041	0.210
Flavonifractor	1.931	-1.301	to	5.164	0.237
Coprococcus	0.657	-0.480	to	1.793	0.241
Intestinibacter	-1.726	-4.725	to	1.272	0.257
Clostridium XVIII	-0.564	-1.553	to	0.425	0.259
Veillonella	-0.323	-0.889	to	0.244	0.264
Intestinimonas	4.771	-3.753	to	13.295	0.264
Oscillibacter	0.958	-0.760	to	2.675	0.272
Bifidobacterium	-0.064	-0.180	to	0.052	0.274
Dialister	0.735	-0.853	to	2.322	0.280
Odoribacter	1.687	-2.058	to	5.431	0.290
Akkermansia	-0.162	-0.537	to	0.214	0.364
Lactobacillus	-0.203	-0.701	to	0.295	0.377
Sutterella	-0.210	-0.740	to	0.320	0.398
Haemophilus	-0.679	-2.399	to	1.042	0.423

Holdemania	12.568	-20.975	to	46.111	0.437
Escherichia/Shigella	-0.218	-0.839	to	0.403	0.439
Parabacteroides	-0.172	-0.681	to	0.337	0.462
Clostridium sensu stricto	-0.219	-0.870	to	0.432	0.492
Fusobacterium	-0.107	-0.439	to	0.224	0.507
Phascolarctobacterium	-0.330	-1.361	to	0.700	0.510
Anaerostipes	0.058	-0.122	to	0.238	0.525
Parasutterella	0.202	-0.599	to	1.003	0.529
Eggerthella	0.858	-2.582	to	4.297	0.530
Gemmiger	0.065	-0.257	to	0.388	0.585
Megasphaera	0.140	-0.645	to	0.924	0.621
Bacillus	-0.649	-4.357	to	3.060	0.625
Actinomyces	-2.312	-17.273	to	12.649	0.680
Blautia	0.031	-0.180	to	0.243	0.692
Alistipes	0.035	-0.215	to	0.285	0.727
Turicibacter	-0.122	-1.059	to	0.815	0.731
Streptococcus	0.035	-0.255	to	0.325	0.762
Prevotella	0.006	-0.068	to	0.081	0.773
Ruminococcus2	-0.013	-0.179	to	0.153	0.783
Roseburia	0.017	-0.207	to	0.241	0.799
Clostridium XlVb	-0.161	-2.937	to	2.615	0.815
Collinsella	0.011	-0.204	to	0.227	0.868
Unclassified	-0.007	-0.188	to	0.175	0.880
Fusicatenibacter	-0.014	-0.442	to	0.414	0.881
Anaerotruncus	-0.784	-26.903	to	25.334	0.909
Faecalibacterium	0.005	-0.153	to	0.162	0.917
Butyricicoccus	0.004	-1.545	to	1.553	0.942

Model 1: Adjusted for age, sex, and BMI.

Abbreviations: BMI, body mass index; CI, confidence interval; SDNN, standard deviation of RR intervals.

Table S4. Multivariate analysis (model 2) of the association between SDNN (ms) and the relative count (%) of gut microbial genera.

**Gut microbial genera**	**Model 2**				
	**β**	**95% CI**			***p*-value**
Lachnospiracea_incertae_sedis	1.449	0.616	to	2.282	0.001

Romboutsia	1.442	0.032	to	2.852	0.045
Bilophila	-3.212	-6.911	to	0.486	0.089
Ruminococcus	0.179	-0.037	to	0.396	0.105
Granulicatella	-80.705	-190.161	to	28.750	0.148
Clostridium XVIII	-0.759	-1.790	to	0.272	0.149
Bacteroides	-0.074	-0.176	to	0.027	0.149
Erysipelotrichaceae_incertae_sedis	-0.511	-1.215	to	0.194	0.155
Clostridium IV	0.149	-0.077	to	0.374	0.197
Flavonifractor	2.076	-1.212	to	5.365	0.216
Saccharibacteria_genera_incertae_sedis	-10.506	-27.402	to	6.390	0.223
Dorea	0.711	-0.461	to	1.882	0.234
Intestinimonas	5.177	-3.477	to	13.831	0.241
Clostridium XlVa	-1.545	-4.191	to	1.101	0.252
Coprococcus	0.659	-0.496	to	1.815	0.263
Veillonella	-0.300	-0.873	to	0.274	0.306
Bifidobacterium	-0.059	-0.177	to	0.058	0.322
Oscillibacter	0.782	-0.959	to	2.524	0.378
Akkermansia	-0.168	-0.557	to	0.222	0.398
Intestinibacter	-1.333	-4.503	to	1.836	0.409
Dialister	0.653	-0.959	to	2.265	0.427
Sutterella	-0.219	-0.761	to	0.323	0.428
Odoribacter	1.541	-2.321	to	5.404	0.434
Anaerostipes	0.071	-0.113	to	0.256	0.448
Haemophilus	-0.644	-2.374	to	1.087	0.466
Phascolarctobacterium	-0.378	-1.430	to	0.674	0.481
Parabacteroides	-0.186	-0.718	to	0.346	0.493
Parasutterella	0.280	-0.533	to	1.094	0.499
Holdemania	11.368	-22.499	to	45.236	0.510
Fusobacterium	-0.109	-0.446	to	0.227	0.523
Escherichia/Shigella	-0.203	-0.835	to	0.429	0.528
Eggerthella	1.077	-2.395	to	4.549	0.543
Clostridium sensu stricto	-0.188	-0.847	to	0.471	0.576
Megasphaera	0.178	-0.626	to	0.982	0.664
Gemmiger	0.063	-0.265	to	0.391	0.705
Actinomyces	-2.879	-18.023	to	12.265	0.709
Bacillus	-0.711	-4.495	to	3.073	0.713

Butyricicoccus	0.293	-1.317	to	1.902	0.721
Streptococcus	0.049	-0.246	to	0.344	0.745
Clostridium XlVb	-0.417	-3.222	to	2.387	0.770
Alistipes	0.037	-0.218	to	0.292	0.776
Roseburia	0.025	-0.203	to	0.252	0.830
Ruminococcus2	-0.018	-0.188	to	0.153	0.840
Faecalibacterium	0.017	-0.148	to	0.181	0.842
Unclassified	-0.014	-0.200	to	0.172	0.883
Blautia	0.016	-0.203	to	0.235	0.887
Lactobacillus	0.022	-0.514	to	0.559	0.935
Prevotella	-0.002	-0.079	to	0.075	0.956
Turicibacter	-0.027	-1.010	to	0.956	0.956
Fusicatenibacter	0.012	-0.426	to	0.450	0.957
Anaerotruncus	-0.559	-26.954	to	25.835	0.967
Collinsella	0.001	-0.219	to	0.221	0.990

Model 2: Adjusted for age, sex, BMI, antidiabetic, antihyperlipidemic, antihypertensive, physical activity (non-winter and winter seasons), smoking, and alcohol drinking.

Abbreviations: BMI, body mass index; CI, confidence interval; SDNN, standard deviation of RR intervals.
